# Supplementary material for: The effects of genital myiasis on the diversity of the vaginal microbiota in female Bactrian camels
Source: BMC Vet Res. 2022 Mar 5;18:87. doi: 10.1186/s12917-022-03189-5 (PMC8897907; doi:10.1186/s12917-022-03189-5)
Supplement: Supplementary file 5 — Additional file 5. [file 12917_2022_3189_MOESM5_ESM.zip › MPL201709200_16s_yy/Treat1/css/js_css/jsPDF-master.zip!/jsPDF-master/examples/canvg_context2d/bar_graph_with_text_and_lines.html]

Bar Graph With Text And Lines


# Bar Graph With Text And Lines

Refresh


SALES


NET\_SALES


01/05/08


01/08/08


02/05/08


02/06/08


02/12/08


03/12/08


04/02/08


04/05/08


04/08/08


04/10/08


05/02/08


06/06/08


07/09/08


08/02/08


08/11/08


09/07/08


10/01/08


10/05/08


12/01/08


120


100


80


60


40


20


0
